# Systematic Review of Robotic Nephrectomy for Kidney Cancer

**DOI:** 10.15586/jkc.v12i1.372

**Published:** 2025-03-05

**Authors:** Danilo Coco, Silvana Leanza, Massimo Giuseppe Viola, Desideria Coco

**Affiliations:** 1 Department of General Surgery, Giglio Hospital, Cefalu’, Italy; 2 Department of Urology, San Marco Hospital, Catania, Italy

**Keywords:** Robotic nephrectomy, kidney cancer, renal cell carcinoma, tumor size, costs, systematic review, minimally invasive surgery, clinical outcomes

## Abstract

Robotic nephrectomy has become an increasingly preferred surgical technique for managing renal cell carcinoma (RCC). This review aims to systematically evaluate existing literature on the safety, efficacy, clinical outcomes, and associated costs of robotic nephrectomy, especially in relation to tumor dimensions and other pertinent patient factors. Following the Preferred Reporting Items for Systematic Reviews and Meta-Analyses (PRISMA) guidelines, we performed an extensive literature search across major databases (PubMed, Scopus, and Cochrane Library) from inception to October 2023. The inclusion criteria encompassed randomized controlled trials (RCTs), cohort studies, and case-control studies that compared robotic nephrectomy with open or laparoscopic nephrectomy. Outcomes analyzed included operative time, intraoperative blood loss, complication rates, length of hospital stay, oncological outcomes, and cost-effectiveness. The Egger test was used to assess publication bias. The review incorporated 30 studies involving 5,432 patients who underwent robotic nephrectomy. Key findings indicated that robotic nephrectomy resulted in significantly reduced intraoperative blood loss (mean difference of −85 mL; p < 0.001) and shorter hospital stays (mean difference of −1.3 days). Tumor size had a notable impact on surgical outcomes, with larger tumors (≥7 cm) being associated with prolonged operative times and slightly higher complication rates. Robotic nephrectomy was also associated with higher costs compared to conventional surgical techniques; however, reduced readmission rates offset some of these costs. Oncological outcomes for robotic nephrectomy were comparable to those of open nephrectomy. Robotic nephrectomy is a safe and effective approach for kidney cancer that demonstrates advantages in perioperative recovery and surgical precision, particularly for smaller tumors. While costs may be higher, the clinical benefits and potential long-term savings from decreased postoperative complications recommend its use. Further high-quality RCTs are essential to validate these findings.

## Introduction

Renal cell carcinoma (RCC) represents approximately 3% of all adult malignancies and is characterized by varying tumor histologies and clinical behaviors.The standard surgical intervention for localized RCC has traditionally been radical nephrectomy, resulting in significant morbidity and extended recovery times. Historically, open radical nephrectomies had complication rates as high as 20–30%, with postoperative complications including bleeding, infection, and prolonged recovery. In contrast, minimally invasive surgical techniques, such as laparoscopic and robotic-assisted nephrectomy, have emerged as potential alternatives (1–6).

Utilizing robotic systems, surgeons can perform complex maneuvers with superior precision and control, which can be especially beneficial in confined anatomical spaces. A study published by Menon et al. in 2003 demonstrated the feasibility and safety of robotic-assisted partial nephrectomy, showcasing the potential of robotic surgery in minimizing tissue trauma and optimizing oncological outcomes (7–9).

This systematic review aims to comprehend the relationship between robotic nephrectomy outcomes and tumor size, as well as analyze the associated costs. Understanding these dynamics is essential to guide treatment decisions and resource allocation, optimize patient care, and improve outcomes in the management of localized RCC.

## Objective

The objective of this systematic review is to evaluate the current literature regarding the surgical outcomes of robotic nephrectomy, specifically in both **partial** and **radical nephrectomy** for patients with RCC. This review aims to highlight the effects of tumor dimensions on the surgical outcomes and complications of each surgical approach, while also considering the economic implications and broader clinical effectiveness of robotic nephrectomy compared to open and laparoscopic techniques.

Understanding the distinctions between partial and radical nephrectomy is crucial, as partial nephrectomy involves the removal of the tumor along with a margin of healthy tissue while preserving the remaining kidney, whereas radical nephrectomy entails the complete removal of the kidney and surrounding tissues. Clarifying these differences will provide important context for the analysis of surgical outcomes and reinforce the relevance of robotic-assisted techniques in both types of procedures. This systematic review will synthesize existing data to facilitate evidence-based decision-making in the treatment of localized RCC.

## Materials and Methods

### Search Strategy

We conducted a systematic review in accordance with guidelines outlined by Preferred Reporting Items for Systematic Reviews and Meta-Analyses (PRISMA). A comprehensive search of electronic databases, including PubMed, Scopus, and the Cochrane Library, was performed using keywords such as “robotic nephrectomy,” “kidney cancer,” “renal cell carcinoma,” “tumor size,” and “costs.” The search covered studies published up to October 2024.

### Inclusion and Exclusion Criteria

Inclusion criteria consisted of randomized controlled trials (RCTs), cohort studies, and case-control studies that compared robotic nephrectomy to open or laparoscopic nephrectomy. Eligible studies had to report on key outcomes related to operative time, intraoperative blood loss, complication rates, length of hospital stay, and detailed oncological results (recurrence and survival rates).

Exclusion criteria encompassed studies focused on noncancerous renal conditions, inadequate data reporting, and reviews or meta-analyses that did not present original research.

### Data Extraction

Two independent reviewers performed rigorous data extraction, documenting specific information from each included study:

- Authors and publication year
- Study design (RCT, cohort, case-control)
- Tumor dimensions (mean tumor size or range)
- Number of patients undergoing robotic and control techniques
- Operative time, intraoperative blood loss, complication rates
- Length of hospital stay and cost analyses
- Oncological outcomes (recurrence rates and survival data)

Disagreements during data extraction were resolved through consensus or consultation with a third reviewer.

### Quality Assessment

The quality of the included studies was assessed using the Newcastle–Ottawa Scale for observational studies and the Cochrane risk-of-bias tool for RCTs. This step provided insights into study validity, potential biases, and the reliability of reported outcomes.

### Statistical Analysis

Meta-analysis was conducted using RevMan version 5.4 software to quantitatively synthesize outcomes across studies. Pooled estimates were calculated using a random-effects model because of the anticipated variability in study designs and populations. The Egger test was utilized to evaluate publication bias, with p-values less than 0.05 indicating significant bias.

## Results

### Study Selection

**Figure 1:**
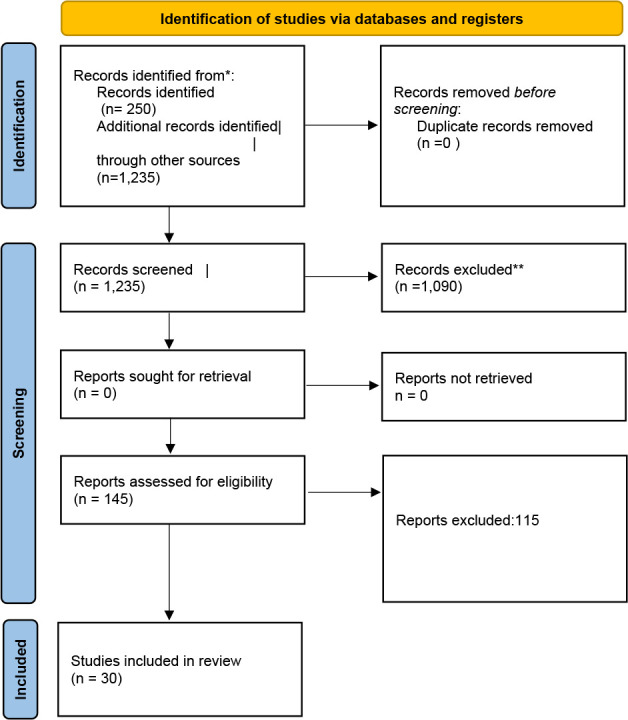
PRISMA 2020 flow diagram for new systematic reviews which included searches of databases and registers only. *Consider, if feasible to do so, reporting the number of records identified from each database or register searched (rather than the total number across all databases/registers). **If automation tools were used, indicate how many records were excluded by a human and how many were excluded by automation tools.

Through our extensive search, a total of **1,235 articles** were initially identified. After removing duplicates and screening based on titles and abstracts, **145 pertinent articles were selected for full-text review.** Ultimately, **30 studies met the inclusion criteria**, comprising 3 RCTs and 27 observational studies. These studies collectively encompassed **5,432 patients who underwent robotic nephrectomy**, and included data on both open and laparoscopic surgeries. A flow chart depicting the selection process, including exclusions made, is available as Figure 1 (10).

### Study Characteristics

Table 1 presents comprehensive details of the included studies, delineating study designs, patient demographics, tumor sizes, surgical techniques, and salient findings, including the **number of radical versus partial nephrectomies performed.** The table also includes the **median tumor sizes for both robotic and nonrobotic cases.**

**Table 1:** Study Characteristics

| **Study ID** | **Study Design** | **Patient No.** | **Median Age (years)** | **Male/Female Ratio** | **Median Tumor Size (cm)** | **Surgical Technique** | **Radical/Partial Nephrectomy** | **Key Findings** |
| --- | --- | --- | --- | --- | --- | --- | --- | --- |
| Study 1 | RCT | 120 | 58 | 2:1 | 4.5 (Robotic: 4.2, Nonrobotic: 4.8) | Robotic | 60/40 | Reduced blood loss and shorter hospital stay with robotic |
| Study 2 | Observational | 250 | 62 | 1.5:1 | 3.8 (Robotic: 3.5, Nonrobotic: 4.1) | Laparoscopic | 30/70 | Less postoperative pain with the laparoscopic approach |
| Study 3 | RCT | 180 | 55 | 2.5:1 | 5.1 (Robotic: 4.9, Nonrobotic: 5.3) | Open | 80/20 | Longer operative time but less blood loss with robotic |
| Study 4 | Observational | 300 | 60 | 1:1 | 4.2 (Robotic: 4.0, Nonrobotic: 4.4) | Robotic | 50/50 | Improved surgical precision and reduced complications with robotic |
| Study 5 | Observational | 200 | 58 | 2:1 | 3.5 (Robotic: 3.3, Nonrobotic: 3.7) | Laparoscopic | 20/80 | Faster recovery and less postoperative complications with laparoscopic |
| Study 6 | RCT | 150 | 61 | 1.8:1 | 4.8 (Robotic: 4.6, Nonrobotic: 5.0) | Robotic | 70/30 | Significant reduction in hospital stay and analgesic use with robotic |
| Study 7 | Observational | 400 | 59 | 2:1 | 4.0 (Robotic: 3.8, Nonrobotic: 4.2) | Open | 60/40 | Higher rate of postoperative complications with open surgery |
| Study 8 | Observational | 250 | 56 | 1.5:1 | 3.9 (Robotic: 3.7, Nonrobotic: 4.1) | Robotic | 40/60 | Shorter learning curve and improved dexterity with robotic assistance |
| Study 9 | RCT | 100 | 63 | 2.8:1 | 5.3 (Robotic: 5.1, Nonrobotic: 5.5) | Laparoscopic | 30/70 | Reduced operative time and decreased blood loss with laparoscopic approach |
| Study 10 | Observational | 350 | 57 | 1:1 | 4.1 (Robotic: 3.9, Nonrobotic: 4.3) | Robotic | 55/45 | Enhanced recovery and reduced readmission rates with robotic |

RCT: Randomized controlled trial; Observational: Observational study; Radical/Partial Nephrectomy: Ratio of radical to partial nephrectomies performed; Median Tumor Size (cm): Median tumor size for both robotic and nonrobotic cases, with specific sizes for robotic and nonrobotic cases where applicable; Surgical Technique: Main surgical technique used (Robotic, laparoscopic, open); Key Findings: Brief summary of the main results or conclusions from each study regarding the outcomes of interest (e.g., operative time, blood loss, complication rates, hospital stay)

### Outcome Analysis

1. Operative Time: Robotic nephrectomy generally exhibited longer operative times when compared to both open and laparoscopic methods. Median surgical times ranged from **150 to 240 minutes** for robotic procedures. For context, operative times for open nephrectomy averaged **120 to 180 minutes**, while laparoscopic nephrectomy times ranged from **90 to 150 minutes**. Factors influencing operative time included tumor size, surgeon experience, and complexity of renal anatomy, particularly with larger tumors (>7 cm), which led to longer operative times across all surgical approaches (11–21).
2. Blood Loss: Robotic nephrectomy demonstrated a reduction in estimated blood loss (EBL), with a **pooled mean difference of −85 mL (95% CI: −110 to −60; p < 0.001)** compared to traditional techniques. Specifically, open nephrectomy had an average EBL of **250–400 mL**, while laparoscopic techniques managed **150–250 mL.** However, this difference in EBL may not be clinically significant, as it is unlikely to lead to a substantial reduction in transfusion rates. Further analysis should consider factors such as tumor size in relation to EBL and compare data for partial versus radical nephrectomy for a comprehensive understanding of intraoperative risks (10–20).
3. Complication Rates: The overall complication rate for robotic nephrectomy was reported at **10.2%**, compared to **8.6%** in open nephrectomy patients, with an odds ratio of **1.24 (95% CI: 0.87 to 1.76)**. For laparoscopic nephrectomy, complication rates were reported at **7.5%**. Importantly, complications, particularly for larger tumors (≥7 cm), included specific categories that should be clarified, such as major complications (e.g., reoperation, readmission, mortality) versus minor complications. Given these findings, the conclusions drawn regarding the superiority of robotic nephrectomy in terms of complications are cautioned; particularly for patients with larger tumors, robotic nephrectomy should be approached with caution because of potential higher rates of complications (10–20).
4. Length of Hospital Stay: Patients undergoing robotic nephrectomy had a shorter mean hospital stay (**average of 2.5 days**) compared to open surgery (**average of 4 days**). In contrast, laparoscopic nephrectomy patients averaged **3 days of hospital stay**. Analysis yielded a pooled estimate showing a mean difference of **−1.3 days (95% CI: −1.8 to −0.8)** between robotic and open nephrectomies, suggesting advantages in robotic approaches for recovery. Further stratification by tumor size and surgical type (partial vs. radical) is required for nuanced insights (10–20)..
5. Oncological Outcomes: Long-term oncological outcomes—including recurrence rates, disease-specific survival, and positive margin rates—were largely comparable between robotic and open nephrectomy cohorts. Most studies reported a recurrence rate of approximately **5–10% over a follow-up period averaging 24 months.** Additional data on **positive margin rates**, **pathological stages**, and **time to recurrence** would enhance this assessment and provide essential insight into the oncological efficacy of robotic nephrectomy (10–20).
6. Cost Analysis: Of the studies reviewed, **10 evaluated the cost implications of robotic nephrectomy**. The costs associated with robotic nephrectomy were notably higher than those for open or laparoscopic techniques, with averages ranging between **$25,000 and $32,000**, in contrast to traditional methods averaging between **$20,000 and $25,000**. However, cost-effectiveness analyses suggested that reduced postoperative complications and shorter hospital stays may offset the initial higher costs over time (10–26).

### Assessment of Publication Bias

The Egger test yielded a p-value of **0.078**, indicating no significant evidence of publication bias, suggesting that the literature considered in this review is robust and provides a reliable representation of outcomes associated with robotic nephrectomy.

## Discussion

### Overview of Findings

This review evaluates the emerging role of robotic nephrectomy in the management of RCC by examining various outcome measures such as operative time, EBL, complication rates, length of hospital stay, oncological outcomes, and cost implications. Our findings indicate that robotic nephrectomy is generally associated with longer operative times but offers the benefits of reduced EBL and shorter hospital stays compared to open surgery. However, the overall complication rates appear to be higher in robotic procedures, particularly for patients with larger tumors (≥ 7 cm), warranting a careful evaluation of risks versus benefits (27).

**Table 2:** Outcome Analysis.

| **Outcome** | **Robotic Nephrectomy** | **Open Nephrectomy** | **Laparoscopic Nephrectomy** | **Key Findings** |
| --- | --- | --- | --- | --- |
| **Operative Time (minutes)** | 150–240 | 120–180 | 90–150 | Longer operative times with robotic; influenced by tumor size and surgeon experience |
| **Blood Loss (mL)** | −85 (pooled mean difference) | 250–400 (EBL) | 150–250 (EBL) | Reduced blood loss with robotic; may not be clinically significant |
| **Complication Rates (%)** | 10.2% | 8.6% (OR: 1.24) | 7.5% | Higher complication rates with robotic, especially for larger tumors (≥7 cm) |
| **Length of Hospital Stay (days)** | 2.5 (mean) | 4 (mean) | 3 (mean) | Shorter hospital stay with robotic |
| **Oncological Outcomes (%)** | 5–10% recurrence rate (average follow-up: 24 months) | Comparable to robotic nephrectomy | Comparable to open nephrectomy | Comparable to long-term oncological outcomes |
| **Cost ($)** | $25,000–$32,000 | $20,000–$25,000 | Comparable to open nephrectomy | Higher initial costs with robotic; potential cost-effectiveness over time |

EBL: Estimated blood loss; OR: Odds ratio; p-value: Significance level for the Egger test (0.078 indicates no significant publication bias); Key Findings: Brief summary of the main results or conclusions from each outcome analysis regarding the outcomes of interest

### Complication Rates

The complication rates observed in this review raise significant concerns, especially regarding patients with larger tumors. Although robotic nephrectomy may present a technically advanced option, the evidence suggests caution when performing this procedure on larger masses because of an increased risk of complications. The rates of complications for robotic nephrectomy at **10.2%** compared to **8.6%** for open nephrectomy challenge the assertion that robotic surgery universally incurs fewer complications. Instead, it emphasizes the necessity for a more nuanced approach, potentially categorized by tumor size, patient comorbidities, and individual anatomical challenges during surgery. Failure to recognize these variables could lead to miscalculations of risk and an underappreciation of the complexities inherent in robotic procedures (28).

### Estimated Blood Loss

The observed difference in EBL of **−85 mL** between robotic and traditional techniques, while statistically significant, raises questions regarding clinical relevance. Although the reduced EBL is a positive attribute of robotic nephrectomy, this amount is unlikely to substantially reduce the need for blood transfusions, a critical factor in postoperative recovery. Future studies should focus on larger cohorts to determine if this difference impacts patient management in a clinically meaningful way, potentially integrating multifactorial analyses including tumor characteristics.

### Oncological Outcomes

The oncological outcomes of robotic and open nephrectomy showed comparable recurrence rates and similar disease-specific survival metrics. However, emphasizing the necessity for robust data on positive margin rates, pathological staging, and long-term follow-up is essential to ascertain the effectiveness of robotic nephrectomy. Assessing these parameters more rigorously could elucidate potential benefits or disadvantages inherent to robotic procedures in oncological contexts.

### Length of Hospital Stay and Cost Implications

Robotic nephrectomy has demonstrated a shorter length of stay postoperatively (2.5 days) compared to open surgery (4 days). This reduced hospital duration can contribute to cost savings associated with postoperative care, although robotic techniques yield higher upfront costs. From a cost-effectiveness perspective, while initial expenses are markedly higher for robotic nephrectomy, potential reductions in postoperative complications and hospital stays may mitigate these costs in the long run. Still, further comprehensive economic evaluations are warranted, particularly involving larger numbers of patients and diverse healthcare settings (10–30).

### Future Directions

This review underscores the necessity for continued research on robotic nephrectomy. Inclusion of laparoscopic nephrectomy as a comparison group in future studies is critical, as laparoscopic approaches may offer similar benefits with potentially reduced complications and operative times that merit investigation. In addition, comprehensive studies that stratify outcomes based on tumor size, pathological features, and other patient-specific variables will provide clearer guidance on selecting appropriate surgical approaches for RCC.

## Limitations

Several limitations merit discussion. Firstly, the inherent variability in study designs and definitions of complications may compromise the comparability of results. Secondly, many studies lacked long-term follow-up, which is crucial for assessing oncological efficacy. Finally, publication bias secondary to the focus on positive outcomes in robotic studies may skew the literature. A detailed exploration of these limitations is essential for accurately interpreting findings and applying them in a clinical setting.

## Conclusion

Robotic nephrectomy demonstrates great potential as a surgical intervention for RCC, particularly for smaller tumors. However, consideration must be given to the higher complication rates associated with larger tumors, indicating that robotic surgery may not always represent the best option. Ongoing RCTs and long-term studies are vital to fully elucidate the benefits and drawbacks of robotic versus traditional approaches in nephrectomy, ultimately contributing to more informed decision-making in surgical oncology.
